# Autophagy-Targeted Vascular Remodeling in Pulmonary Arterial Hypertension: Molecular Mechanisms and Therapeutic Perspectives

**DOI:** 10.3390/jcdd13080387

**Published:** 2026-08-13

**Authors:** Miao Li, Limei Piao

**Affiliations:** 1 Department of Cardiology and Hypertension, Yanbian University Hospital, Yanji 133000, China; 18632992019@163.com; 2 Jilin Provincial Key Laboratory of Stress and Cardiovascular Disease, Yanbian University, Yanji 133000, China

**Keywords:** PAH, pulmonary arterial hypertension, autophagy, AMPK/mTOR, mitophagy, vascular remodeling, therapeutic targets

## Abstract

Pulmonary arterial hypertension (PAH) is a severe cardiovascular disease characterized by progressively increased pulmonary vascular resistance and right heart failure. Its pathogenesis involves multiple factors, including genetic predisposition, inflammation, oxidative stress, and imbalances between cell proliferation and apoptosis. Recent studies indicate that autophagy has a context-dependent dual role in PAH. Flux-competent autophagy may be protective by clearing damaged mitochondria, limiting excessive inflammation, and maintaining metabolic homeostasis, whereas excessive autophagy initiation or impaired autophagosome-lysosome degradation may promote metabolic dysfunction, inflammatory signaling, abnormal vascular cell phenotypes, and pulmonary vascular remodeling. This focused narrative review summarizes the molecular mechanisms and key signaling pathways linking autophagy to PAH, with emphasis on PTEN-induced kinase 1 (PINK1)/Parkin-mediated mitophagy and the AMP-activated protein kinase (AMPK)/mechanistic target of rapamycin (mTOR) energy-sensing axis. It also evaluates potential therapeutic strategies targeting key nodes of autophagy, such as AMPK activators and mTOR inhibitors, along with their clinical research progress. Finally, this review provides an outlook on future research directions, emphasizing the need to further elucidate the dynamic regulatory mechanisms and cell-type specificity of autophagy in order to advance the clinical translation of autophagy-targeted precision therapies for PAH.

## 1. Introduction

Pulmonary arterial hypertension (PAH) is a severe cardiovascular disease that increases pulmonary vascular resistance, leading to right ventricular overload and, in severe cases, right heart failure and death. Recent Global Burden of Disease 2021 estimates demonstrate that PAH remains an important cause of morbidity and mortality worldwide and that China contributes substantially to the global disease burden [1]. A recent systematic review has further summarized the epidemiology and healthcare burden of PAH in China [2].

**Figure 1:**
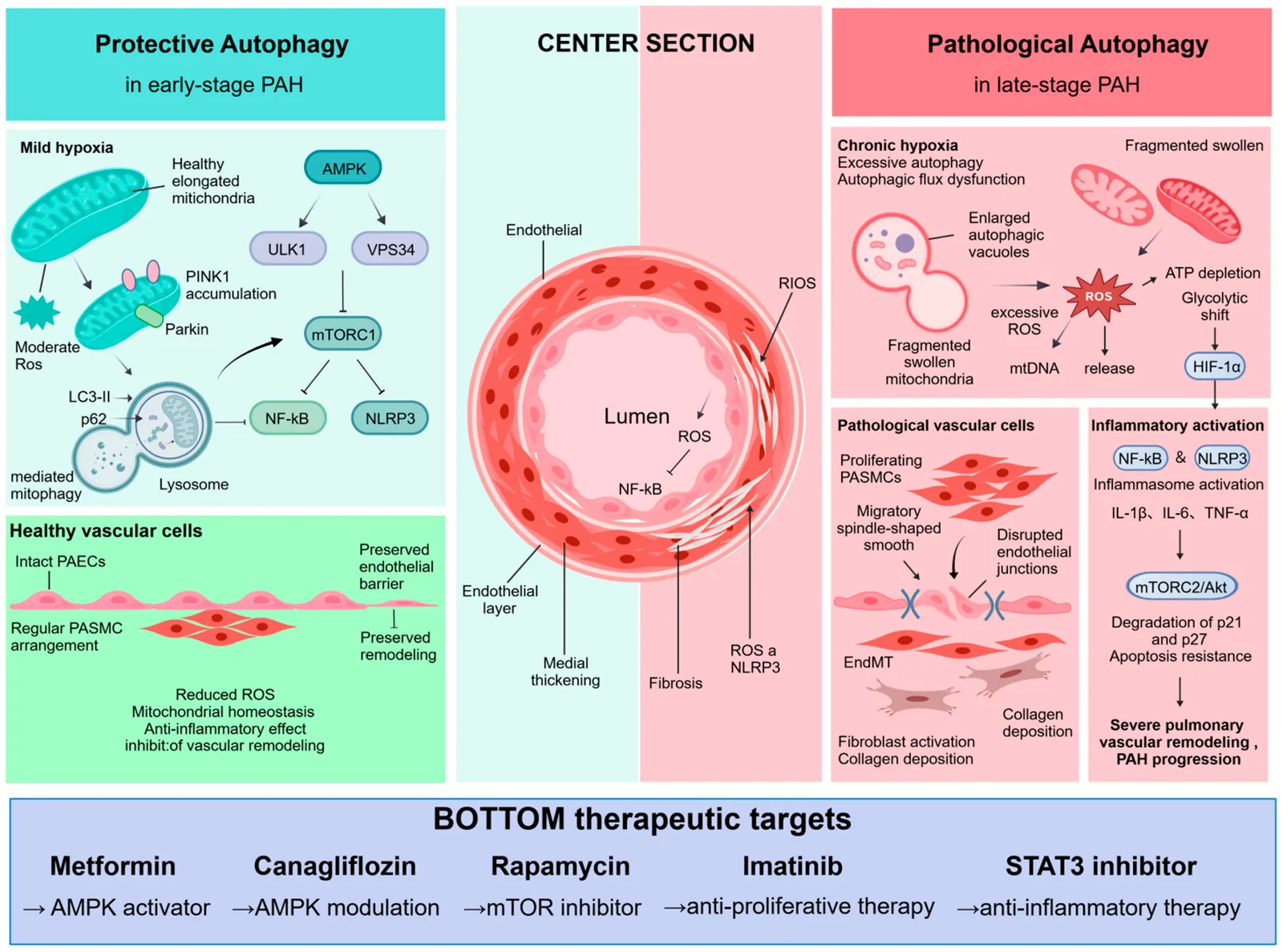
Context-dependent dual roles of autophagy in pulmonary arterial hypertension (PAH). In early-stage PAH, moderate, flux-competent autophagy and PINK1/Parkin-mediated mitophagy clear damaged mitochondria, reduce reactive oxygen species and inflammatory signaling, preserve pulmonary artery endothelial cell barrier integrity and pulmonary artery smooth muscle cell homeostasis, and limit vascular remodeling. In advanced PAH, excessive or dysregulated autophagy and impaired autophagic flux promote mitochondrial injury, adenosine triphosphate depletion, glycolytic/hypoxia-inducible factor1alpha reprogramming, mitochondrial DNA release, NF-κB/NLRP3 activation, endothelial-to-mesenchymal transition, smooth muscle cell proliferation and apoptosis resistance, fibroblast activation, collagen deposition and pulmonary vascular remodeling. Representative autophagy-related strategies include AMPK modulation with metformin or canagliflozin, mTOR inhibition with rapamycin, and inhibition of proliferative or inflammatory pathways with imatinib or STAT3 inhibitors. The stage distinction is schematic and context dependent. Abbreviations: AMPK, AMP-activated protein kinase; mTOR, mechanistic target of rapamycin; NF-κB, nuclear factor kappa B; NLRP3, NLR family pyrin domain-containing 3; PINK1, PTEN-induced kinase 1; STAT3, signal transducer and activator of transcription 3. The schematic illustration created with BioGDP.com.

PAH involves a complex pathogenesis, with a primary pathophysiological mechanism being structural and functional changes in the pulmonary vasculature, such as abnormal proliferation of pulmonary artery endothelial cells and smooth muscle cells [3]. Autophagy is a process of cellular degradation and recycling of proteins and organelles to maintain cellular homeostasis. Typically, autophagy plays a protective role in cells, but disruption of the autophagic mechanism or excessive autophagy often leads to cell death [4]. Based on recent research findings, we have identified a close relationship between autophagy and PAH. Autophagy can play both protective and detrimental roles in PAH by regulating cellular homeostasis, inflammation, and metabolism. This article introduces the mechanisms of action of autophagy in PAH, related signaling pathways, therapeutic targets, and future research directions. The context-dependent dual roles of autophagy and the principal therapeutic targets discussed in this review are illustrated in Figure 1.

### Literature Search Strategy

This narrative review was based on searches of PubMed/MEDLINE and Europe PMC for English-language articles published from January 2020 to July 2026; earlier landmark studies were included when essential for mechanistic context. Search terms included combinations of “pulmonary arterial hypertension,” “autophagy,” “mitophagy,” “PINK1,” “Parkin,” “AMPK,” “mTOR,” “NF-κB,” “MAPK,” “BMPR2,” “genetics,” and “vascular remodeling.” Original mechanistic studies, animal studies, clinical investigations, and directly relevant reviews were considered, while duplicate reports, studies unrelated to pulmonary vascular disease, and articles without clear mechanistic or clinical relevance were excluded. Reference lists of key articles were also screened manually. Because this was a narrative review, no formal meta-analysis or risk-of-bias assessment was performed.

## 2. The Dual Role of Autophagy in Pulmonary Arterial Hypertension: Protection and Injury

### 2.1. Protective Role of Autophagy

In the pathological progression of PAH, the regulatory role of autophagy exhibits significant stage dependence. Under mild or transient stress, particularly during early disease, moderate autophagy may protect cells and tissues through the following mechanisms:

#### 2.1.1. Clearance of Damaged Mitochondria (Mitophagy)

Moderate autophagy clears damaged mitochondria, reduces excessive reactive oxygen species (ROS) generation, thereby mitigating oxidative stress-induced injury to pulmonary artery endothelial cells (PAECs) and pulmonary artery smooth muscle cells (PASMCs), and delaying vascular remodeling [5]. The PTEN-induced kinase 1 (PINK1)/Parkin pathway is a key quality control mechanism mediating the selective clearance of damaged mitochondria, i.e., mitophagy [6]. Following mitochondrial depolarization, PINK1 stabilizes on the outer mitochondrial membrane, recruiting and activating the E3 ubiquitin ligase Parkin. Parkin then extensively ubiquitinates outer mitochondrial membrane proteins, marking damaged organelles for clearance. By targeted removal of dysfunctional mitochondria, this pathway directly reduces the primary source of excessive ROS, thereby alleviating oxidative stress and its downstream harmful effects. By maintaining mitochondrial network integrity and metabolic function, PINK1/Parkin-mediated mitophagy helps preserve cellular energy metabolism and redox homeostasis [7]. In the pathological context of PAH, the normal functioning of this pathway is protective. It limits oxidative damage, thereby inhibiting the pathological phenotypic transformation of pulmonary vascular cells. Therefore, a functional PINK1/Parkin-mediated mitophagy pathway is an important mechanism counteracting the vascular remodeling process.

In certain cases, mitophagy can also be initiated through Parkin-independent mechanisms, such as receptor-mediated processes involving BCL2-interacting protein 3-like (BNIP3L/NIX). BNIP3L/NIX is an outer mitochondrial membrane protein that interacts with the autophagy-related protein 8 (ATG8) family and recruits autophagosomes. Through its LC3-interacting region (LIR), BNIP3L/NIX binds microtubule-associated protein 1 light chain 3 (LC3), directly linking damaged mitochondria to autophagosomes and initiating mitophagy [8].

#### 2.1.2. Inhibition of Inflammatory Response

Moderate autophagy can modulate the nuclear factor kappa B (NF-κB) pathway, the NLR family pyrin domain-containing 3 (NLRP3) inflammasome, and mitogen-activated protein kinase (MAPK) signaling, thereby limiting the release of pro-inflammatory cytokines, including interleukin-1β (IL-1β), interleukin-6 (IL-6), and tumor necrosis factor-α (TNF-α). Autophagy interacts with inflammatory signaling pathways, affecting the inflammatory response in PAH [9]. NF-κB is a central transcription factor in inflammation, regulating the expression of various pro-inflammatory cytokines. Autophagy can inhibit NLRP3 inflammasome activation by clearing damaged mitochondria and suppressing ROS generation [10]. Autophagy can also inhibit inflammatory responses by regulating other signaling pathways, such as AMP-activated protein kinase (AMPK) and MAPK.

### 2.2. Disease-Promoting Role of Autophagy

Although early autophagy activation can exert protective effects by clearing damaged organelles and inhibiting excessive proliferation, persistent or dysregulated autophagy during advanced disease may exacerbate vascular remodeling and organ damage through multi-dimensional mechanisms. This “double-edged sword” effect primarily stems from imbalances in autophagic flux, cell-type-specific responses, and abnormal interactions within the metabolic-inflammatory network.

#### 2.2.1. Autophagosome Accumulation and Metabolic Dysregulation

In advanced PAH, autophagosome accumulation may reflect increased autophagosome formation, impaired lysosomal clearance, or both; therefore, increased autophagic vacuoles alone should not be interpreted as evidence of increased autophagic flux. This process can cause excessive degradation of organelles (e.g., mitochondria, endoplasmic reticulum) and key functional proteins, directly disrupting cellular energy metabolism and homeostasis. For example, overactivation of mitophagy in pulmonary artery smooth muscle cells (PASMCs) can lead to substantial loss of functional mitochondria, reducing intracellular adenosine triphosphate (ATP) synthesis capacity to 40–50% of normal levels, forcing cells to shift to glycolytic energy production (i.e., the Warburg effect) [11]. This metabolic reprogramming is continuously amplified via the AMPK/mechanistic target of rapamycin complex 1 (mTORC1) signaling axis: AMPK activation inhibits mTORC1 activity, relieving the phosphorylation-dependent inhibition of the autophagy initiation complex unc-51-like kinase 1 (ULK1), further driving autophagosome formation. Simultaneously, glycolytic intermediates (e.g., lactate) stabilize hypoxia-inducible factor 1α (HIF-1α), creating a vicious cycle of hypoxia signaling and metabolic abnormality, supporting the abnormal proliferation and migration of PASMCs [12]. Notably, metabolic reprogramming not only provides biosynthetic precursors required for proliferation but can also induce histone acetylation through accumulated acetyl-CoA, promoting epigenetic activation of pro-fibrotic genes, including collagen type I alpha 1 chain (COL1A1) and alpha-smooth muscle actin (α-SMA) and accelerating adventitial fibrosis [13].

#### 2.2.2. Driving Pathological Inflammation

Overactivated autophagy can also amplify pathological damage through inflammatory signaling cascades. Mitochondrial dysfunction can lead to ROS bursts, further inducing the release of mitochondrial DNA into the cytosol, thereby activating the NLRP3 inflammasome and promoting the maturation and secretion of the inflammatory cytokines IL-1β and interleukin-18 (IL-18) [14]. Furthermore, autophagosome membrane components such as LC3-II can activate the NF-κB signaling pathway via Toll-like receptors TLR4/9, inducing sustained release of pro-inflammatory factors like TNF-α and IL-6, creating a vascular inflammatory microenvironment. This inflammatory response not only directly damages the endothelial barrier function but can also activate pericytes and fibroblasts through paracrine mechanisms, promoting excessive extracellular matrix deposition, ultimately leading to irreversible occlusive vasculopathy [15].

#### 2.2.3. Cell Type-Specific Injury

The disease-promoting effects of autophagy exhibit significant heterogeneity among different pulmonary vascular cells. In pulmonary artery endothelial cells (PAECs), dysregulated autophagy may compromise endothelial homeostasis and barrier integrity. Endothelial-to-mesenchymal transition (EndMT) has been identified in remodeled pulmonary vessels from patients with PAH and experimental pulmonary hypertension and has been implicated in endothelial dysfunction and vascular remodeling [16]. Conversely, in PASMCs, overactivation of autophagy relieves proliferation arrest by degrading cell cycle inhibitors such as p21 and p27, and enhances cell survival via the mTORC2/Akt signaling axis, endowing PASMCs with an ‘apoptosis-resistant’ characteristic. Single-cell transcriptomic analysis has revealed that the expression levels of autophagy-related 5 (ATG5) and Beclin 1 (BECN1) in PASMCs from patients with advanced PAH are 2–3 times higher than in patients with early-stage disease and show a significant positive correlation with Ki-67 (proliferation marker) expression [17]. The contrasting protective and disease-promoting effects of autophagy across PAH pathological contexts are summarized Table 1.

## 3. Key Signaling Pathways and Mechanisms of Autophagy

### 3.1. PINK1/Parkin Pathway

The PINK1/Parkin pathway is a core regulatory mechanism of mitophagy, responsible for recognizing and clearing damaged mitochondria, thereby maintaining mitochondrial quality and cellular homeostasis. Under normal physiological conditions, PINK1 can be imported into the inner mitochondrial membrane, cleaved by presenilin-associated rhomboid-like protein (PARL) in the intermembrane space into a 52 kDa fragment, and subsequently degraded by the proteasome [18]. However, when mitochondria are damaged (e.g., membrane potential depolarization), PINK1 cannot enter the inner membrane, leading to the stable accumulation of full-length PINK1 on the outer mitochondrial membrane, where it is activated through autophosphorylation at Ser228. Activated PINK1 then phosphorylates ubiquitin at Ser65. This phosphorylated ubiquitin acts as a key signal, recruiting cytosolic Parkin (an E3 ubiquitin ligase) to the outer mitochondrial membrane. Subsequently, PINK1 phosphorylates Parkin at Ser65, activating its ubiquitin ligase activity. Activated Parkin then ubiquitinates outer mitochondrial membrane proteins (e.g., voltage-dependent anion channel 1 (VDAC1), mitofusin 1 (MFN1), and mitofusin 2 (MFN2)), forming polyubiquitin chains. These ubiquitinated proteins serve as ‘eat-me’ signals recognized by autophagic receptor proteins including sequestosome1 (SQSTM1/p62) and optineurin (OPTN). The autophagic receptors bind to the LC3 protein on the nascent autophagosomal membrane, anchoring the damaged mitochondrion to the forming autophagosome. Finally, the autophagosome engulfs the damaged mitochondrion and fuses with a lysosome, where degradation occurs by lysosomal hydrolases [19].

Studies have shown that in the early stages of PAH, moderately activated autophagy is accompanied by increased PINK1 expression, which in turn upregulates the PINK1/Parkin pathway, promoting the clearance of damaged mitochondria and alleviating pulmonary vascular remodeling [20]. Therefore, in-depth exploration of the mechanisms and regulatory modes of the PINK1/Parkin pathway is of great value for developing therapeutic strategies for related diseases.

### 3.2. AMPK Signaling Pathway

The AMPK pathway is a key regulator of cellular energy metabolism. AMPK is a heterotrimeric complex composed of a catalytic α subunit and two regulatory β and γ subunits. All possible combinations generate 12 different AMPK complexes. The α subunit has two isoforms, α1 and α2, encoded by genes PRKAA1 and PRKAA2, respectively, and contains a conserved Ser/Thr kinase domain where threonine 172 (Thr172) is a critical phosphorylation site for its activity. The α subunit also contains an autoinhibitory domain and regions for binding the β and γ subunits. The β subunit has two isoforms, β1 and β2, encoded by PRKAB1 and PRKAB2, respectively, and contains a glycogen-binding domain and regions for binding other subunits, potentially participating in AMPK binding to targets like glycogen synthase. The γ subunit has three isoforms, γ1, γ2, and γ3, encoded by PRKAG1, PRKAG2, and PRKAG3, respectively, and contains four cystathionine-β-synthase tandem repeats forming two Bateman domains, each capable of binding one AMP or ATP molecule [21]. Its regulatory mechanisms can be divided into direct and indirect regulations.

#### 3.2.1. Direct Regulation

AMPK precisely activates the autophagic pathway through multi-site phosphorylation of core regulatory factors in the initiation phase of autophagy. During autophagy initiation, AMPK phosphorylation of ULK1 is particularly crucial. It targets multiple key residues on ULK1 (e.g., Ser467, Ser555, Thr574, Ser637), where phosphorylation of Ser555 and Ser637 directly enhances ULK1 kinase activity, while phosphorylation of Ser467 and Thr574 provides a dual activation mechanism by relieving the inhibitory effect of mTORC1 on ULK1. Activated ULK1 then phosphorylates downstream autophagy-related proteins (e.g., ATG13 and FIP200), promoting the recruitment of ATG protein complexes to membrane regions such as endoplasmic reticulum-mitochondria contact sites, initiating the nucleation of the pre-autophagosomal structure and forming the initial isolation membrane scaffold [22]. Furthermore, AMPK regulation of vacuolar protein sorting 34 (VPS34), the core catalytic subunit of the class III phosphatidylinositol 3-kinase complex is a key step in autophagosomal membrane elongation. AMPK directly phosphorylates VPS34 at Thr163 and Ser165, exerting a dual function: selectively inhibiting non-autophagic VPS34 complexes involved in vesicular trafficking, while enhancing the stability of pro-autophagic VPS34 complexes containing ATG14L and increasing their capacity to synthesize phosphatidylinositol 3-phosphate (PI3P). PI3P, as a crucial lipid signaling molecule, recruits FYVE or PX domain-containing proteins (e.g., WD repeat domain phosphoinositide-interacting protein 2 (WIPI2) and double FYVE domain-containing protein 1 (DFCP1)) to the isolation membrane, thereby mediating membrane elongation and curvature, ultimately leading to autophagosome closure. Notably, AMPK regulation of the VPS34 complex exhibits spatiotemporal specificity, as its phosphorylation can dynamically influence the complex’s localization to subcellular structures, enriching it at autophagosome formation hotspots such as endoplasmic reticulum-mitochondria contact sites [23].

AMPK coordinately regulates the rate and scale of autophagosome generation through a dual targeting mechanism activating ULK1 and reprogramming VPS34 function. Particularly under energy stress conditions, AMPK rapidly initiates ULK1-mediated autophagic signals and simultaneously optimizes the lipid kinase activity of the VPS34 complex, ensuring efficient autophagosome generation to cope with the metabolic crisis. These mechanisms not only reveal the direct pathways of AMPK in autophagy regulation but also provide a theoretical basis for targeting the AMPK-ULK1/VPS34 axis in the treatment of autophagy-related diseases.

#### 3.2.2. Indirect Regulation

As a core regulator of cellular energy homeostasis, AMPK also participates in autophagy activation through indirect regulatory mechanisms, key among which is the dual inhibition of the mTORC1 signaling pathway. On one hand, AMPK phosphorylates RPTOR (regulatory-associated protein of mTOR), a structural component of mTORC1, directly disrupting the stability of this complex. On the other hand, AMPK activates TSC2 (tuberous sclerosis complex 2), enhancing its interaction with Ras homolog enriched in brain (Rheb) GTPase, thereby blocking mTORC1 membrane localization and activation. Since mTORC1 is a key negative regulator of autophagy, inhibiting its activity relieves the suppression of the ULK1/ATG1 kinase complex, initiating autophagosome nucleation and elongation [24].

### 3.3. mTOR Signaling Pathway

mTOR is an evolutionarily conserved serine/threonine protein kinase belonging to the phosphatidylinositol 3-kinase-related kinase (PIKK) family. It plays a critical regulatory role in cell growth, metabolism, proliferation, survival, and autophagy. Specifically, mTOR can form two distinct multi-protein complexes: mTORC1 and mTORC2 [25]. Among these, mTORC1 is a major regulator of cell growth and metabolism, responding to growth factors, nutrients, energy status, and stress signals. For example, it regulates protein synthesis, lipid synthesis, and specifically inhibits autophagy by suppressing the autophagy initiation complex ULK1. During this process, under nutrient deprivation or low energy conditions, AMPK is activated and phosphorylates TSC2, thereby inhibiting mTORC1 activity and promoting autophagy [26]. Conversely, growth factors and hormones activate the phosphoinositide 3-kinase (PI3K)/protein kinase B (Akt) pathway, inhibiting the TSC1/TSC2 complex, thereby activating mTORC1 and suppressing autophagy. Similarly, amino acids translocate mTORC1 to the lysosomal surface via Rag GTPases and the Ragulator complex for activation, ultimately achieving inhibition of autophagy [27]. On the other hand, mTORC2 is primarily involved in cell survival, cytoskeletal regulation, and metabolic processes. Notably, the activation mechanism of mTORC2 is relatively complex. Recent studies suggest it can phosphorylate and fully activate Akt, which in turn can activate mTORC1 [28]. Importantly, the functions of these two mTOR complexes are particularly critical in pathological states; the pro-proliferative and pro-survival effects of Akt/mTOR have been demonstrated in human idiopathic PAH patients and animal PAH models [29]. Studies have also shown that mTORC2 indirectly activates mTORC1 in an AMPK-independent manner by phosphorylating Akt jointly promoting cell proliferation and survival [30], suggesting that both mTOR complexes play a pathogenic role in the vascular remodeling of PAH.

### 3.4. NF-κB, MAPK, and BMPR2 Signaling in Autophagy-Related PAH

NF-κB, MAPK, and bone morphogenetic protein receptor type 2 (BMPR2) signaling form an interconnected regulatory network linking autophagy to inflammation and pulmonary vascular remodeling. By removing damaged mitochondria and limiting mitochondrial ROS and mtDNA release, intact autophagic flux can reduce NF-κB-dependent inflammatory priming and NLRP3 inflammasome activation [9,14]. Conversely, persistent MAPK signaling may promote maladaptive autophagy: in PASMCs, high-mobility group box 1 activates extracellular signal-regulated kinases 1/2 (ERK1/2) and dynamin-related protein 1 (Drp1), increasing mitochondrial fission and autophagy, reducing BMPR2/inhibitor of DNA binding 1 (Id1) signaling, and promoting proliferation, migration, and vascular remodeling [31]. BMPR2 itself can undergo autophagy-associated lysosomal degradation in human PAECs and PASMCs, while BMPR2 haploinsufficiency may further enhance autophagy, suggesting a feed-forward mechanism that amplifies PAH progression [32].

### 3.5. Genetic Susceptibility, Autophagy, and PAH

Genetic susceptibility may influence PAH partly by altering mitochondrial quality control and endolysosomal homeostasis. BMPR2 variants are the most known cause of heritable PAH, and large-scale sequencing studies have also implicated genes such as SOX17, AQP1, and GDF2 [33]. The clearest direct connection with autophagy involves BMPR2, because reduced BMPR2 dosage can increase autophagy and facilitate further lysosomal loss of BMPR2 protein [32]. In addition, reduced endothelial expression of the endolysosomal Ras-related protein 7 (RAB7) GTPase has been identified in PAH; experimental RAB7 deficiency impairs endosome-lysosome function and autophagy, promotes mitochondrial dysfunction and endothelial senescence, and induces pulmonary hypertension [17]. These observations support the concept that genetic defects and autophagic dysfunction may interact, although direct autophagy-related mechanisms remain to be established for many PAH-associated genes.

## 4. Potential Therapeutic Strategies Targeting Autophagy in PAH

The role of autophagy in PAH exhibits stage dependence and cell type specificity. Current therapeutic approaches mostly involve modulating key nodes of autophagy.

### 4.1. AMPK Activators: Metabolic-Autophagic Dual Regulation

Experiments have shown that canagliflozin, a sodium–glucose cotransporter 2 (SGLT2) inhibitor with mild sodium–glucose cotransporter 1 (SGLT1) inhibitory activity, has a direct impact on PASMCs. It can alleviate pulmonary artery and right ventricular remodeling, as well as the right ventricular hypertrophy index and fibrosis degree, in monocrotaline (MCT)-induced PAH rats by activating the AMPK pathway. It has also been confirmed that under hypoxic conditions, canagliflozin inhibits PASMC proliferation [34]. Another study exploring structure–activity relationships around a specific pyridin-2-one scaffold identified a series of novel AMPK inhibitor lead compounds, CHEMBL3780091. Compound **13a** was observed to effectively inhibit AMPK activity and exhibit favorable anti-proliferative activity against PASMCs. Furthermore, 13a significantly reduced right ventricular systolic pressure, attenuated vascular remodeling, and improved right heart function in hypoxia-induced PAH rats [35]. Additionally, studies have shown that AMPK prevents disease progression in hypoxia-induced PAH, and metformin has been found to improve pulmonary vascular remodeling by activating the AMPK pathway [36]. The mechanism of metformin in treating PAH has now entered a Phase II clinical trial [37]. This trial enrolled 20 patients, with 19/20 reaching the target dose and all completing the study protocol. Results showed no clinically significant lactic acidosis or changes in oxidative stress markers. Although metformin did not change the 6 min walk distance, there was a significant improvement in the right ventricular area fractional change. This demonstrates that metformin treatment was safe and well-tolerated in this study of PAH patients. Further exploratory analysis suggests metformin may be associated with improved right ventricular area change [38].

### 4.2. mTOR Inhibitors: Restoring Autophagic Homeostasis

The mechanism of action of mTOR inhibitors in treating PAH is complex, centering on inhibiting the mTOR pathway to restore cellular autophagic homeostasis, thereby intervening in the vascular remodeling process [39]. Research by Shi et al. indicates that the mTOR inhibitor rapamycin specifically inhibits mTORC1 signaling upon short-term (6 h) treatment of human PASMCs, but prolonged exposure (>24 h) inhibits both mTORC1 and mTORC2 activity, potentially weakening its anti-proliferative effect. However, combining rapamycin with the platelet-derived growth factor receptor (PDGFR) inhibitor imatinib more effectively inhibited PASMC proliferation and significantly reduced pulmonary artery pressure, right ventricular hypertrophy, and vessel wall thickening in both cell models MCT- or Sugen 5416/hypoxia (SuHx)-induced PAH rat models [40]. Another study found that in an MCT-induced PAH rat model, the combination of the mTOR inhibitor rapamycin and the signal transducer and activator of transcription 3 (STAT3) inhibitor HO-3867 more comprehensively inhibited phosphorylated Akt (p-Akt; downstream of mTORC2) and p-STAT3 in lung tissue, as well as phosphorylated ribosomal protein S6 (p-S6; downstream of mTORC1) and p-STAT3 in right ventricular tissue, thereby more effectively improving hemodynamic parameters and pathological changes compared to monotherapy [41].

In conclusion, future therapeutic strategies should move beyond single-node inhibition of mTOR. They should focus on synergistically blocking multiple pathogenic signals through combination therapy (e.g., with imatinib or STAT3 inhibitors) and performing precise interventions based on disease stage and specific cellular signaling contexts, to more effectively achieve the restoration of pulmonary vascular homeostasis. Representative autophagy-related intervention strategies are summarized in Table 2.

## 5. Conclusions

Autophagy is a key compensatory mechanism that cells employ in response to stress. Its core function is to maintain cellular homeostasis, enhance cell survival under adverse conditions, and, under specific circumstances, induce programmed cell death. In PAH, autophagy plays a complex dual role. Appropriate autophagic activity helps protect the vascular endothelial barrier, maintain normal vascular tone, alleviate inflammatory responses, and inhibit abnormal phenotypic switching of pulmonary vascular wall cells. Conversely, insufficient or overactivated autophagy can induce persistent pulmonary vasoconstriction, microthrombus formation, exacerbated inflammatory infiltration, and excessive proliferation of vascular wall cells, thereby accelerating pulmonary vascular remodeling and PAH progression. Currently, the regulatory role of autophagy in PAH has been preliminarily recognized, and clinical studies are beginning to identify therapeutic strategies and drugs that exert efficacy by modulating autophagy. However, due to the inherent complexity of the autophagic process, its regulatory effects on specific pathological changes remain controversial. In the future, with deeper exploration of the molecular mechanisms and signaling pathways related to autophagy in PAH, targeting autophagy may become an important direction for treating PAH and its related diseases.

## Figures and Tables

**Table 1:** Potential roles of autophagy in different pathological stages of PAH.

Pathological Dimension	Protective Role of Moderate Autophagy	Disease-Promoting Role of Autophagy Imbalance	Representative Pathways/Molecules
Mitochondrial homeostasis	Clearance of damaged mitochondria, reduction of ROS, maintenance of redox homeostasis	Excessive or aberrant mitophagy leads to loss of functional mitochondria, promotes glycolytic reprogramming	PINK1/Parkin, BNIP3L/NIX, LC3, p62
Inflammatory response	Limits leakage of mtDNA and ROS, inhibits NLRP3 inflammasome activation	Sustained activation of inflammasome and NF-κB, promotes release of IL-1β, IL-6, TNF-α	NLRP3, NF-κB, MAPK
Vascular cell phenotype	Maintains endothelial barrier and cellular homeostasis, limits abnormal phenotype switching	Promotes EndMT, PASMCs proliferation/migration, apoptosis resistance, and adventitial fibrosis	AMPK/mTOR, Akt/mTORC2, HIF-1α
Autophagic flux status	Coordination of autophagosome formation and lysosomal degradation, maintains substrate turnover	Uncoupling of autophagosome generation and degradation; static markers may be elevated but function not necessarily enhanced	LC3-II, Beclin-1, p62, lysosomal function

**Table 2:** Potential intervention strategies targeting autophagy-related pathways.

Intervention Direction	Representative Drugs/Strategies	Main Mechanisms of Action	Existing Evidence	Issues Requiring Attention
AMPK modulation	Metformin, canagliflozin, AMPK inhibitor candidates	Metabolic reprogramming, autophagy initiation, inflammatory response	Animal experiments and early clinical studies suggest potential benefits	AMPK effects are cell- and stage-dependent
mTOR inhibition	Rapamycin	Relieves mTORC1-mediated inhibition of ULK1, suppresses pro-proliferative signaling	Cell and animal models show attenuation of vascular remodeling	Long-term use may affect mTORC2; safety needs evaluation
Combination therapy	Rapamycin + imatinib; rapamycin + STAT3 inhibitor	Simultaneously blocks pro-disease networks including mTOR, PDGFR, and STAT3	Greater improvement compared with monotherapy in animal models	Clinical translation requires clarification of dosage, toxicity, and target population
Precise regulation of autophagic flux	Improves the coupling between autophagosome formation and degradation	Lysosomal function modulation, mitochondrial quality control targets	Still primarily mechanistic research	Reliable in vivo evaluation systems for autophagic flux need to be established

## Data Availability

No new data were created or analyzed in this study. Data sharing is not applicable to this article.
